# Deletion of chromosome 17 as a novel cytogenetic finding in chronic neutrophilic leukemia: A case report

**DOI:** 10.3892/ol.2013.1235

**Published:** 2013-03-07

**Authors:** YUANYUAN CHEN, SHUYE WANG, WEI WANG

**Affiliations:** Department of Hematology, First Affiliated Hospital of Harbin Medical University, Harbin, Heilongjiang 150001, P.R. China

**Keywords:** chromosome, chronic neutrophilic leukemia, deletion, marker

## Abstract

Chronic neutrophilic leukemia (CNL) is an infrequently encountered myeloproliferative disorder characterized by sustained neutrophilic leukocytosis, hepatosplenomegaly and the absence of the Philadelphia chromosome. This study presents a 60-year-old male patient with a diagnosis corresponding to the WHO classification criteria for CNL who was treated at the First Affiliated Hospital of Harbin Medical University (Harbin, China). Chromosome analysis of the patient’s bone marrow cells showed 44, XY, -17, -17 in all 20 metaphase spreads. Homoharringtonine was used to control the patient’s high white cell count for a short time, although after two weeks, the patient complained of left upper quadrant discomfort and his blood count showed a white blood cell count of 40.8×10^9^/l. However, the patient continued to undergo maintenance therapy and was discharged in good clinical condition with hydroxyurea treatment for 31 months. Usually, patients with CNL have normal karyotypes and no specific cytogenetic abnormalities have been identified. To the best of the authors’ knowledge, this is the first case in the literature of a patient exhibiting the deletion of chromosome 17 with CNL. We concluded that deleted of tumor suppressor genes located on 17p13.1, such as p53, may be associated with the development of CNL. Patients with CNL have a poor prognosis, although the present patient has exhibited a prolonged stable phase with oral chemotherapy drug treatment.

## Introduction

Chronic neutrophilic leukemia (CNL) is a rare disorder characterized by a persistently elevated leukocyte count due to an increase in the number of mature neutrophils with a high neutrophil alkaline phosphatase (NAP) score, raised serum levels of vitamin B12 and uric acid, the absence of the Philadelphia (Ph) chromosome and hepatosplenomegaly, when a situation capable of inducing a reactive leukemoid reaction, such as underlying infectious disease or neoplasia, does not exist (1). These features, together with the lack of eosinophilia, basophilia or monocytosis, and the absence of BCR-ABL transcripts, distinguish CNL from chronic myeloid leukemia (CML), atypical CML and chronic myelomonocytic leukemia, as defined by the French-American-British (FAB) Cooperative Group (2). Unlike CML, no characteristic clonal chromosomal or molecular markers have been confirmed. The present study reports a case of CNL involving the deletion of chromosome 17, which, to the best of the authors’ knowledge, has not been previously reported. The study was approved by the Ethics Committee of the First Affliated Hospital of Harbin Medical University, Harbin, China. Written informed consent was obtained from the patient’s son.

## Case report

**Figure 1:**
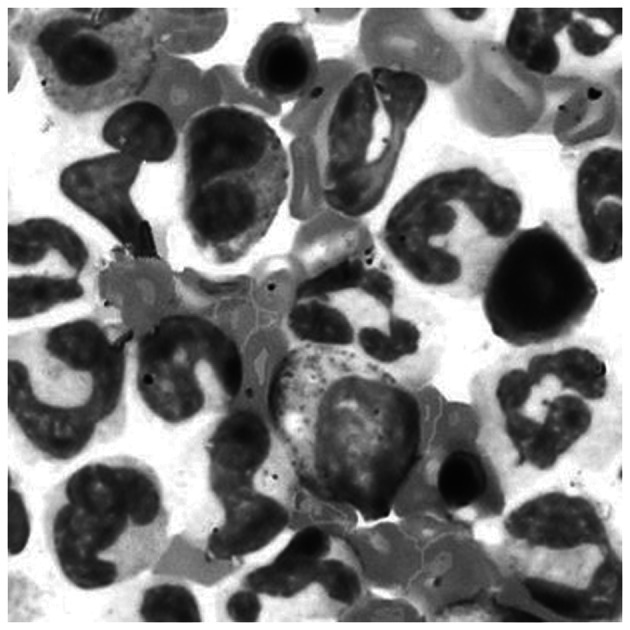
Wright-Giemsa-stained slide of an aspirated bone marrow specimen showing marked myeloid hyperplasia which was mainly due to mature neutrophilic expansion. Magnification, ×400.

**Figure 2:**
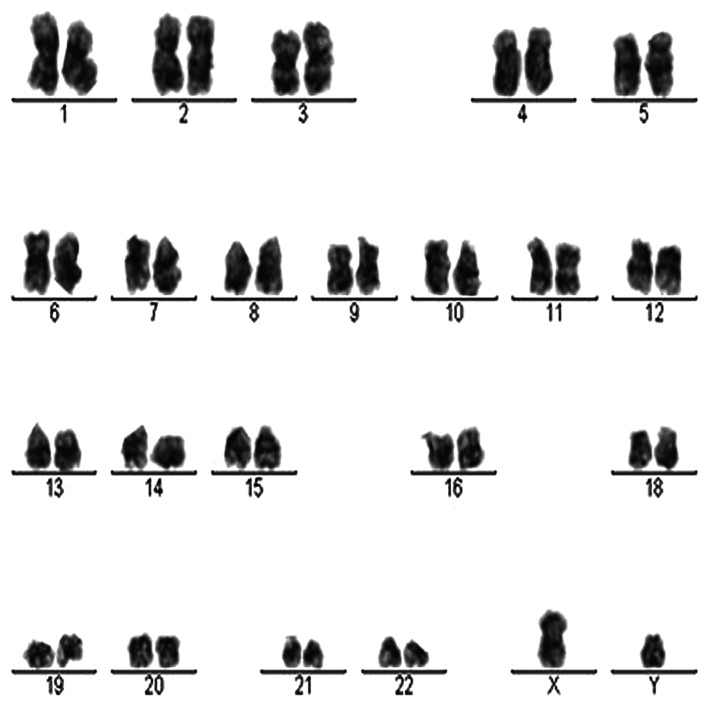
G-banded bone marrow metaphase spread and karyotype showing the deletion of chromosome 17.

A 61-year-old male patient presented a history of upper abdomen heaviness and fatigue for one month in April 2010. All systems appeared normal when examined, with the exception of an enlarged spleen 5 cm below the costal margins with hardening, clear boundaries, poor mobility and no tenderness. A complete blood count showed hemoglobin at 136 g/l and a leukocyte count of 52.1×10^9^/l, with a differential count of 79% stab and segmented neutrophils and 21% lymphocytes. The platelet count was 100×10^9^/l. An aspirated bone marrow specimen was extremely hypercellular with marked myeloid hyperplasia which was predominantly mature neutrophilic expansion (Fig. 1). The myeloid-erythroid ratio was 4.5/1. Biochemical analyses were normal with the exception of significant increases in serum vitamin B12 (2,000 pg/ml), creatinine (108.8 *μ*mol/l) and neutrophil alkaline phosphatase (NAP) (361 U/l; normal range 180–250 U/l). Molecular genetic analysis did not reveal somatic mutations in the JAK2 and BCR-ABL fusion genes. Chromosome karyotype analysis showed a result of 42, XY, -17, -17 in all 20 metaphase spreads (Fig. 2), while the Ph chromosome was not detected. Thus, the patient was diagnosed with CNL associated with the absence of chromosome 17. The patient was discharged and received 3 mg homoharringtonine daily for 15 days. The neutrophil count gradually declined to a normal level and physical examination results were normal with no splenomegaly. Two weeks later, the patient complained of left upper quadrant discomfort. The patient’s spleen had enlarged to 12 cm below the left costal margin and his white blood cell count increased to 40.8×10^9^/l. The patient has since been undergoing maintenance therapy with hydroxyurea. In addition, the patient’s leukocyte count has been stabilized at ≤30×10^9^/l. At present, the patient has remained well on subsequent follow-up visits for 31 months since the initial diagnosis of CNL.

## Discussion

Despite CNL being first reported in 1920 (3), the steering committee of the World Health Organization (WHO) Classification of Neoplastic Diseases only acknowledged CNL as a distinct myeloproliferative disorder in 2001 (4). The clinical and laboratory findings of the patient in the present study corresponded to the WHO classification criteria for the diagnosis of CNL (5), so a diagnosis of CNL was made. The disease appears to predominantly afflict older individuals with a 2:1 male to female ratio and an overall median survival of 30 months (6). The clinical course is heterogeneous, with a definite risk of mortality due to blastic transformation (4).

The clonal nature of CNL has been the subject of controversy (7) as it may be heterogenous from case to case (8). Cytogenetic studies have identified various karyotypic abnormalities in CNL to date, including a missing chromosome 2 replaced by a marker chromosome (9), trisomy 8 (10), trisomy 21 (11), trisomy of chromosome 9 and deletion 20(q) (12), deletion of 11q23 (13) and deletion of the entire long arm of chromosome 20 (14). The present patient exhibited the deletion of chromosome 17 in CNL, which has not been reported previously. Human chromosome 17 has been implicated in a wide range of human genetic diseases (15). For example, the tumor suppressor gene p53 is located on 17p13.1. Herrera *et al*(16) reported that aneuploidy of chromosome 17 and deletion at the 17p13.1 locus of the TP53 gene were genetic alterations observed often in solid tumors. p53 somatic alterations are detected in ∼50% of human cancers (17), including cancer of the colon, stomach, breast and esophagus. These findings suggest that deleted tumor suppressor genes, such as p53 in chromosome 17, may be associated with the development of CNL.

The present patient exhibited a chromosomal abnormality which has not previously reported in connection to CNL. At present, R banding and conventional karyotyping analysis have identified numerous cases of numerical and structural abnormalities of chromosome 17, including chromosome 17 polysomy in metastatic breast cancer (18), isochromosome 17q in acute promyeloblastic leukemia (19), ring 17 chromosome in flecked retina (20), prenatal diagnosis of trisomy 17 mosaicism (21) and monosomy 17 in CML (18). Consequently, chromosome 17 is notable among the human chromosomes in a number of respects.

A standard therapy for CNL remains to be defined. The successful use of splenic irradiation, splenectomy, interferon-alpha and oral cytoreductive agents, such as busulfan and hydroxyurea, aid in the control of the high white cell counts and splenomegaly, but are by no means curative. Bone marrow transplantation may be an option for younger patients with a suitable donor (22). The present patient refused to undergo a bone marrow transplant due to his economic status and old age. The patient appeared to have a steadily poorer response to treatment with homoharringtonine. However, the patient has exhibited a prolonged stable phase with oral hydroxyurea treatment, although there is a definite risk of mortality from leukemic transformation or progressive, refractory neutrophilic leukocytosis.

Although the WHO classification reports a variable prognosis, there are certain studies suggesting that the progression of CNL is extremely rapid and the prognosis is poor (23,24). Three-quarters of patients succumb to the disease within two years, mainly due to cerebral bleeding (25), the pathogenesis of which remains unclear. In summary, abnormalities in chromosome 17 occur infrequently in leukemia and to the best of the authors’ knowledge, paired deletion involving chromosome 17 has not been previously reported in CNL patients. Further studies are necessary to investigate the clonality, molecular pathogenesis and optimal therapy of CNL.
